# Photo‐Cross‐Linked Dual‐Responsive Hollow Capsules Mimicking Cell Membrane for Controllable Cargo Post‐Encapsulation and Release

**DOI:** 10.1002/advs.201600308

**Published:** 2016-12-12

**Authors:** Xiaoling Liu, Dietmar Appelhans, Qiang Wei, Brigitte Voit

**Affiliations:** ^1^ Leibniz‐Institute für Polymerforschung Dresden e.V.Hohe Straße 6D‐01069DresdenGermany; ^2^ Organic Chemistry of PolymersTechnische Universität DresdenD‐01062DresdenGermany

**Keywords:** controllable release, hollow capsules, pH and temperature stimulus, post‐encapsulation

## Abstract

Multifunctional and responsive hollow capsules are ideal candidates to establish highly sophisticated compartments mimicking cell membranes for controllable bio‐inspired functions. For this purpose pH and temperature dual‐responsive and photo‐cross‐linked hollow capsules, based on silica‐templated layer‐by‐layer approach by using poly(*N*‐isopropyl acrylamide)‐*block*‐polymethacrylate) and polyallylamine, have been prepared to use them for the subsequent and easily available post‐encapsulation process of protein‐like macromolecules at room temperature and pH 7.4 and their controllable release triggered by stimuli. The uptake and release properties of the hollow capsules for cargos are highly affected by changes in the external stimuli temperature (25, 37, or 45 °C) and internal stimuli pH of the phosphate‐containing buffer solution (5.5 or 7.4), by the degree of photo‐cross‐linking, and the size of cargo. The photo‐cross‐linked and dual stimuli‐responsive hollow capsules with different membrane permeability can be considered as attractive material for mimicking cell functions triggered by controllable uptake and release of different up to 11 nm sized biomolecules.

## 1 Introduction

Mimicking morphologies and properties of cell compartment with artificial building blocks have attracted intense research interest in the field of bioinspired self‐assembled structures.^1^ The design of artificial cell membrane was among the first subject to be studied^2^, ^3^, ^4^, ^5^, ^6^, ^7^, ^8^ for selective uptake and release of (bio)molecules and particles as key characteristics of cellular compartments controlling intra‐ and intercellular signaling processes.

One of the first bioinspired functions of artificial cell membrane to be investigated in self‐assembled systems was the ability to respond to internal stimuli such as the presence of biomolecules and enzymes,^9^, ^10^, ^11^, ^12^, ^13^, ^14^, ^15^ redox conditions,^16^, ^17^, ^18^ or ionic strength^19^ and pH^20^, ^21^, ^22^, ^23^ of the environment and external stimuli such as light^24^, ^25^ or temperature^26^, ^27^ to control the transport behaviors. Most of these “smart” systems developed were used to elicit cargo release by a single stimulus.^28^

One appealing strategy that has emerged recently is the introduction of polymeric nanoparticle systems that can respond to multiple stimuli associated with pathological conditions^29^ and thereby assuring cargo release in biomedical applications. However, there is only a limited number of literature reports available so far describing polymeric hollow capsules^28^, ^30^, ^31^ and polymersomes^32^ that can modulate drug release in response to the combinations of dual stimuli such as pH/temperature,^26^, ^33^ pH/reduction,^30^ temperature/reduction,^32^ and enzyme/enzyme.^28^, ^34^ Most of these described hollow capsule systems were degradable by redox potential^30^, ^32^ and in the presence of enzymes,^28^, ^34^ while their pH and temperature response states were far from those present in cellular compartments.^26^, ^33^ Thus, there is still a lack of multi‐responsive hollow capsules with a tunable membrane permeability that can control both, the post‐encapsulation of biomolecules in the nanometer (nm) range of up to 11 nm under physiological conditions, and their sustained release triggered by stimuli being compatible with biological processes in cells.

**Scheme 1:**
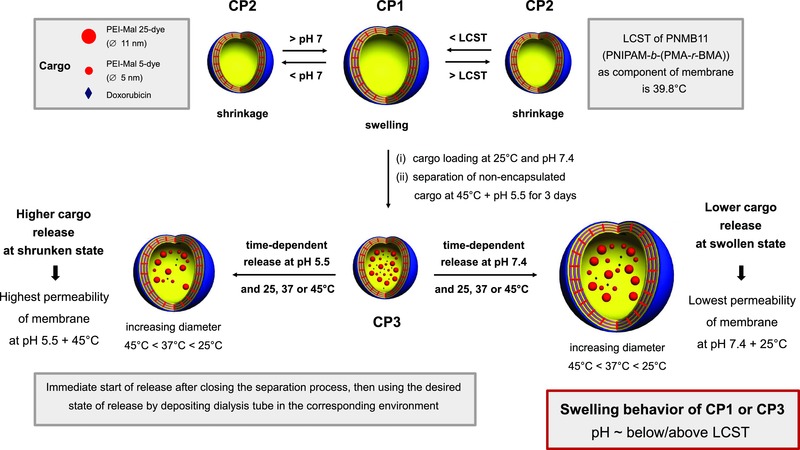
Dual stimuli‐responsive behavior of photo‐cross‐linked hollow capsule CP1 (based on [PAH/PNMB11]_3_/PAH/PSS multilayers) upon temperature and pH changes and the dual‐responsive controlled release characteristics of cargos with different sizes across the membrane of photo‐cross‐linked hollow capsules CP3. Release of cargo carried out at pH 7.4 in PBS environment and at pH 5.5 in phosphate buffer environment. Generally, location of cargo (macro)molecules is in the cavity (shown), but also in the membrane/shell of the hollow capsules (not presented here).

Multi‐responsive hollow capsules have been reported as ideal candidates mimicking cell compartments,^35^, ^36^ nanoreactors,^31^, ^37^, ^38^ or multicompartmentalized entities,^39^, ^40^, ^41^, ^42^, ^43^ for example, demonstrating a successful exchange of educts and products to feed enzymes inside and outside of those supramolecular assemblies. For most of these systems reactive enzymes as well as nanoparticles were enclosed into the inner cavity of the polymeric hollow capsules during their formation process,^31^, ^35^, ^36^, ^37^, ^40^ However, only very limited studies exist for the post‐encapsulation under physiological conditions of small molecules and nm‐sized proteins and polysaccharides into preformed nanocapsules.^26^, ^44^, ^45^, ^46^ Cell membranes are characterized by highly controlled and selective uptake as well as release of various biomacromolecules and thus, this feature has still to be incorporated into the membrane of polymeric hollow capsules for mimicking more realistic cell compartments. Thus, the overall goal of this study is to show that complementary processes of uptake and release by/from specifically designed polymeric hollow capsules are possible at physiological pH and in phosphate buffer at pH 5.5, as well as at different temperatures (**Scheme** **1**). This kind of schizophrenic behavior is needed in future multicompartmentalized systems for use in synthetic biology, thus nanocapsules may uptake specific biologically active molecules, but at the same time can also release other biomolecules.

Polyelectrolyte capsules with cross‐linked membrane, obtained via the layer‐by‐layer (LbL) deposition of polymers on colloidal template particles and subsequent removal of the core particles,^47^, ^48^ are attractive candidates for encapsulation of a diverse range of low molecular weight and macromolecules species^49^ allowing control over the capsule wall thickness, permeability, and stability.^30^ In order to preserve the spherical shape of hollow capsules under varying conditions mostly chemical cross‐linking^30^, ^50^, ^51^, ^52^, ^53^ was used. UV irradiation has been reported as an alternative way to facilitate the design and fabrication of pH‐stable and responsive polymersome nanoreactors, for example, for tuning the switch on and off state of (multi)enzymatic reactions.^54^, ^55^ But this technique was so far not exploited for the stabilization of multiresponsive polyelectrolyte capsules.

We recently have reported photo‐cross‐linked pH‐responsive polymersomes using 2‐hydroxy‐4‐(methacryloyloxy) benzophenone (BMA) as an effective photo‐cross‐linker requiring only short and mild UV irradiation.^56^ Motivated by its simplicity this photo‐cross‐linkable BMA was used in the present study to facilitate the fabrication of stable pH and temperature dual stimuli‐responsive hollow capsules using the LbL assembly of suitable polyelectrolytes onto silica core templates^47^, ^48^ (Scheme S1, Supporting Information) and thereby designing a new type of polymeric capsules to be potentially triggered by stimuli in the tumor lesion and in synthetic biology. For the LbL assembly approach new dual‐responsive photo‐cross‐linkable block copolymers poly‐(*N*‐isopropyl acrylamide)‐*block*‐poly[methacrylic acid‐*co*‐2‐hydroxy‐4‐(methacryloyloxy) benzophenone] (PNMB) were realized as the anionic polyelectrolyte to build the hybrid polyelectrolyte multilayer system together with cationic poly(allylamine hydrochloride) (PAH). Compared to other thermo‐ and pH‐responsive systems reported in the literature,^26^, ^52^, ^57^, ^58^, ^59^ the PNMB structure has the important advantage to be both thermo‐ and pH‐responsive, and in addition, contains photo‐cross‐linkable groups. We could show that the resulting hollow capsules not only possess a robust covalent‐stabilized structure due to the photo‐cross‐linking but also exhibit tunable membrane permeability which provides good stimuli‐responsive (pH and temperature) retain and release behavior for small molecules such as doxorubicin (Dox) and for larger macromolecules, in this case rhodamine B labeled, maltosylated poly(ethyleneimine), PEI‐Mal (5K) and PEI‐Mal (25K), which can be considered as models for proteins. One of the major goals of this study was to show that our stimuli‐responsive hollow capsules can be easily used for cargo post‐encapsulation at physiological pH and room temperature (Scheme 1), avoiding, for example, any annealing steps as known for capsules with micrometer thick membranes.^26^, ^44^

## 2 Result and Discussion

### 2.1 Block Copolymer Synthesis and Characterization

**Figure 1:**
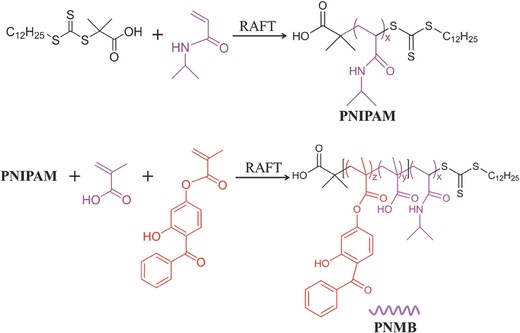
General procedure for the synthesis of block polymer PNMB.

In order to fabricate capsules with desired dual‐responsive properties for temperature and pH, one has to design and synthesize suitable polymer materials. We synthesized a dual‐responsive and photo‐cross‐linkable block copolymer PNMB via reversible addition‐fragmentation chain‐transfer radical polymerization (RAFT) using a two‐step approach (**Figure** **1**. In brief, starting from a RAFT chain transfer agent (CTA) and NIPAM forming the thermo‐sensitive block PNIPAM as macro‐CTA, methacrylic acid (MA) and benzophenone methacrylate (BMA) have been statistically copolymerized in different ratios as second block. The ratio between NIPAM and MA was 2:1, 1:1, 1:2, and 1:4, denominated as PNMB21, PNMB11, PNMB12, and PNMB14, while BMA was incorporated with 5 mol% in the MA polymer chain. In addition, a 1:1 random copolymer PRMNB was also prepared by RAFT for comparison. The chemical composition, molecular weights, and dispersity (*Ð*) of the various PNMB block copolymers and random copolymer PRMNB are presented in **Table** **1** and the polymers were characterized by ^1^H NMR spectroscopy and gel permeation chromatography (GPC) (Table 1 and Figure S1, Supporting Information). Furthermore, there was an almost linear relationship between macro‐CTA/monomer ratio and molecular weights for all four samples and the molecular weight distributions always remained narrow (*Ð* ≤ 1.30). Hence, the low dispersity and the good agreement of copolymer composition and molecular weights with the monomer feed ratio indicated a controlled manner of the polymerization process. The resulting copolymers with appropriate molecular weights (below 20 000 g mol^−1^) are ideal candidates for fabricating polyelectrolyte multilayers because of their good solubility in buffer and having enough carboxyl groups to form polyelectrolyte multilayers using LbL technique. Thus, these functionalized photo‐cross‐linkable PNMB block copolymers enabled us to realize the above‐mentioned aim of capsules with dual‐controllable membrane functions (Scheme 1).

**Table 1:** Molecular parameters of block copolymers and random copolymer synthesized by RAFT

Polymer	Feed ratio of NIPAM and MA [mmol]	Ratio of NIPAM and MAa)	BMAb) [mol%]	*M*_n_ c) [g mol^−1^]	*M*_w_ d) [gmol^−1^]	*Đ* e)
PNMB21	6.67:3.33	2:1	5	13 400	16 800	1.26
PNMB11	5:5	1:1	5	13 000	15 900	1.23
PNMB12	3.33:7.33	1:2	5	12 600	14 900	1.19
PNMB14	2:8.6	1:4	5	12 200	14 200	1.17
PRMNB	5:5	1:1	5	13 200	16 100	1.22

^a,b)^ Calculated by ^1^H NMR spectroscopy

^c–e)^ Obtained from GPC.

For checking the thermo‐ and pH‐sensitivity of the synthesized block copolymers, the lower critical solution temperature (LCST) behavior of PNMB in dependence of the pH values were investigated by turbidity measurements and those are summarized in Figures S2–S4 (Supporting Information) and shortly discussed in the Supporting Information part. From the analysis of turbidity study, PNMB11 was selected as an ideal candidate for fabricating polyelectrolyte multilayers because of its LCST (39.8 °C) being higher than the nominal body temperature in the physiological environment (pH 7.4). In addition, its LCST is lower than the nominal body temperature at slightly acidic environment (Figure S4, Supporting Information) and with a composition of 1:1 (NIPAM:MA) it contains enough carboxyl groups to form effectively polyelectrolyte multilayers using LbL technique. The transmittance of block copolymer PNMB11 was also investigated for reversibly switching over several cycles between 25 and 45 °C (Figure S2B, Supporting Information) and impressively, reversible volume phase transition behavior over five heating–cooling cycles was found, and therefore all following investigations are focused on PNMB11. Thus, the LCST of the block polymer could be accurately tuned by adjusting the fraction of NIPAM units in the block copolymer chains and pH of the environment. Such key features in those dual‐responsive block copolymers could be used to design and fabricate multi‐responsive capsules with tunable LCST.

### 2.2 Multilayers Assembly on Planar Substrates

**Figure 2:**
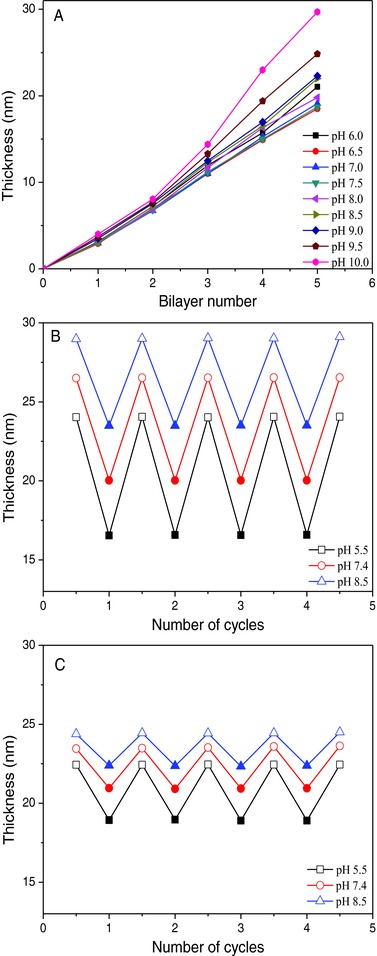
A) Effect of pH on multilayers thickness on planar surface as a function of bilayer number. Reversible swelling‐shrinking of multilayer films [PAH/PNMB11]_3_/PAH/PSS on planar substrate, photo‐cross‐linked for B) 0 and C) 30 min, upon switching between temperature 25 and 45 °C at pH 5.5, pH 7.4, and pH 8.5 buffer, respectively (open symbol = 25 °C; solid symbol = 45 °C) (*n* = 3). The maximal standard deviations of (A)–(C) are ±1.05, ±0.62, and ±0.48, respectively.

To check LbL multilayers formation with the cationic poly(allylamine hydrochloride) (PAH, molecular weight 70 000 g mol^−1^) and the anionic PNMB11 polyelectrolytes, consecutive LbL assembly on planar substrates was performed with an alternate bilayer PAH/PNMB11, followed by the formation of a capping layer PAH/PSS in analogy of the later described formation of the hollow capsules. There, the capping layer PAH/PSS was used to prevent cross‐linking during the formation process of the individual hollow capsules. This multilayer fabrication was monitored by ellipsometric measurements. Suitable pH conditions for preparing a multilayer comprising PAH and PNMB11 were investigated. The effective charge of the polymer under different pH conditions represents a crucial parameter in the LbL technique, since electrostatic interactions are the main driving force governing the assembly process of the polyelectrolytes used in our study, which was supported by zeta potential measurements of the polyelectrolytes PAH and PNMB11 (Figure S5, Supporting Information). To simulate the formation of stable and permeable hollow capsules with membrane thickness of about 20–40 nm, first evaluation focused on the formation of multilayers on planar substrates with the deposition of at least 4–6 bilayers consisting of PAH and PNMB11. For that, the substrate was alternatively brought into contact with the solutions of PAH and PNMB11, and by that a regular and continuous film buildup was demonstrated (**Figure** **2**A). Using pH values between 6.5 and 8 and adsorbing five bilayers on substrate very thin films with thickness in the range of 20 nm were formed.

In addition, to study the dependence of the surface properties of photo‐cross‐linked multilayers on temperature, corresponding multilayers were treated with UV light for 30 min. Thus, the stability of non‐cross‐linked and cross‐linked multilayer films was investigated when the temperature was reversibly switched for several cycles between 25 and 45 °C (Figure 2B,C and Figure S6, Supporting Information). In essence, all multilayers show the desired swelling/shrinking behavior triggered by the pH and temperature stimuli, while the calculation of the swelling ratios of the multilayers gives some additional quantitative information about (Table S1, Supporting Information) the pH‐ and temperature‐dependency of multilayers (discussed below). First, this is caused by the protonation or deprotonation of the carboxylic acid segment of the PNMB block copolymers at low and high pH solutions, respectively. On the other hand, the thermo‐responsive PNIPAM segments of the PNMB block copolymers extend or coil at low and high temperature, respectively. Moreover, this swelling/shrinking behavior of multilayers can be additionally tailored by controlling the degree of cross‐linking (Figure 2C and Figure S6, Supporting Information). Thus, non‐cross‐linked multilayers always outlined a higher swelling power in different pH buffer than their photo‐cross‐linked counterparts, but also the non‐cross‐linked multilayers retained their good temperature and pH sensitivity over four cycles of swelling/shrinking indicated by their changes in thickness (Figure 2B), similar as found for the photo‐cross‐linked multilayers (Figure 2C). In addition, low pH triggered larger swelling for multilayers than high pH, and the larger swelling was observed below LCST at high pH whereas above LCST at low pH the largest shrinking of the multilayers was observed. Additionally, analyzing swelling ratios, calculated for above and below LCST, also confirms the above‐mentioned conclusions (Table S1, Supporting Information).

Furthermore, partial cross‐linked multilayers (Figure S6A, Supporting Information: 10 min irradiation) showed a good swelling power relative to multilayers irradiated longer (Figure 2C). However, multilayers irradiated for 20 min and longer were even more cross‐linked and consequently demonstrated a low swelling power and exhibited a low degree of temperature and pH sensitivity (Figure 2C and Figure S6B, Supporting Information).

These findings highlight the strong influence of the photo‐cross‐linking time on the swelling power of the cross‐linked multilayers. In summary, the PAH/PNMB11 multilayers simultaneously possess good temperature and pH sensitivity and the degree of dual‐responsivity can be further controlled by the cross‐linking time.

### 2.3 Multilayers Assembly onto Colloidal Particles

**Figure 3:**
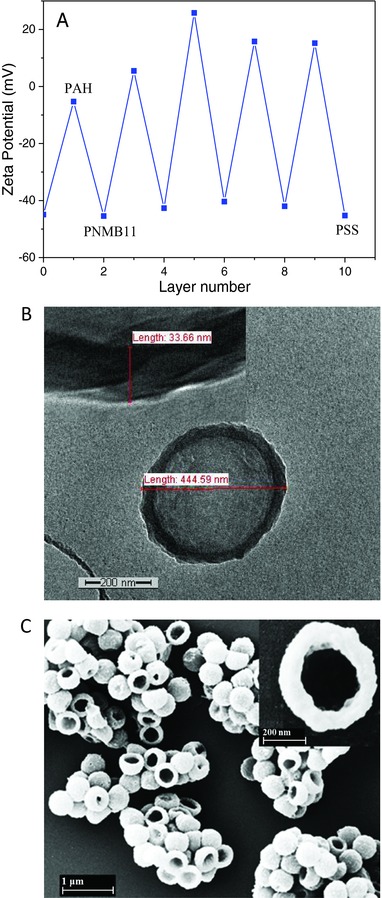
A) Zeta potential as a function of layer number for the [PAH/PNMB11]_4_/PAH/PSS‐coated silica particles. B) TEM and C) SEM images of [PAH/PNMB11]_3_/PAH/PSS‐coated capsules, photo‐cross‐linked for 30 min (prepared using 500 nm diameter SiO_2_ particles). The insets show membrane thickness of individual capsules (B) and a single capsule at higher magnification (C).

Using the approach as implemented on the planar substrates, the [PAH/PNMB11]_3_/PAH/PSS) multilayers were deposited on spherical silica particles (500 nm) to fabricate the photo‐cross‐linked capsules with dual pH and temperature sensitivity. As outer bilayer PAH/PSS was assembled acting as protective capping bilayer to prevent the photo‐cross‐linking of the capsules with each other (Scheme S1, Supporting Information). To prove the successful formation of multilayer on silica particles, the variation in the zeta potential of silica particles coated with the bilayer PAH/PNMB11 was investigated as a function of the number of layers (**Figure** **3**A). The initial zeta potential of the silica surface changed from −45 to −2 mV after the adsorption of cationic PAH polymer. This value is still negative, since the particle surface was not completely covered by PAH polymer. This means that the adsorbed PAH had not enough positive charge to offset the negative charge of bare silica particles. However, the increase of about 40 mV as compared with the bare particles was sufficient to start the fabrication of the desired hollow capsules. The subsequent addition of the anionic PNMB11 led again to a change in the surface charge to −45 mV. This negative zeta potential obtained enabled us further to carry out subsequent adsorption of the positively charged PAH. Such alternating reversal in the sign of the zeta potential is characteristic of LbL assembly of polyelectrolyte multilayers on particles surface, suggesting a successful stepwise layer growth of PAH and PNMB11.

Following our concept (Scheme S1, Supporting Information), a cross‐linking step was applied to ensure the integrity of capsules after the removal of the template making use of the well‐established photo‐cross‐linker BMA integrated in PNMB11 (Figure 1).^60^ The exposure of the [PAH/PNMB11]_3_/PAH/PSS multilayers deposited on the silica particles under the unfiltered light of a mercury lamp for 30 min gave the desired cross‐linked multilayers on the surface of silica particles. By subsequent etching out the silica template by NH_4_F/HF buffer at pH 7.5, hollow capsules were smoothly obtained. In order to confirm the shape of hollow capsules, transmission electron microscopy (TEM) and scanning electron microscopy (SEM) measurements were performed to observe the morphology of the capsules prepared by [PAH/PNMB11]_3_/PAH/PSS multilayer. From the TEM images (Figure 3B) the regular spherical shape of the capsules with uniform diameters of about 445 nm can be clearly observed. Also the magnified image (Figure 3B, inset) clearly revealed the existence of a cross‐linked multilayer shell of the capsules with a thickness of about 33 nm. Generally, a shell thickness of 30 ± 5 nm for the hollow capsules was determined by TEM. Furthermore, the collapsed structure, shown by the SEM images in Figure 3C, indicated that the individual hollow capsules retain their diameters. These diameters are similar to those obtained by TEM study after the removal of the silica template. Due to the vacuum drying needed for carrying out SEM measurements, some of the hollow capsules with their flexible multilayer shell were not stable enough to sustain the hollow spherical structure. This led to the partial rupture of the vesicular structure of the hollow capsules. Also the magnified image (Figure 3C, inset) clearly demonstrates the existence of hollow capsules with uniform diameters of about 440 nm.

In the following, we determined the best conditions to achieve hollow capsules which are on the one hand stable over a longer time, but on the other hand, should be able to show a significant swelling power upon stimuli, which needs a fine‐tuning of the bilayer numbers and the cross‐linking density. As illustrated in Figure S7 (Supporting Information), some ruptures or degradation of the non‐cross‐linked hollow capsules took place (Figure S7A, Supporting Information), while the hollow capsules did not completely disassemble. However, the capsules retained more and more their diameter and spherical shape after removal of the template with increasing the UV‐irradiation periods (5, 10, 20, 30, and 50 min). Corresponding TEM images are shown in Figure S7B–F (Supporting Information). Especially after 30 min of UV irradiation the spherical shape of the hollow capsules is preserved when [PAH/PNMB11]_3_/PAH/PSS multilayers were used. However, the diameter of the capsules is reduced upon further UV irradiation for 50 min (Figure S7F, Supporting Information) due to increased cross‐linking density. Thus, one can state that UV irradiation for 30 min is enough to remain the spherical shape of the capsules providing partially cross‐linked capsules, hopefully, with a relatively high degree of swelling.

**Figure 4:**
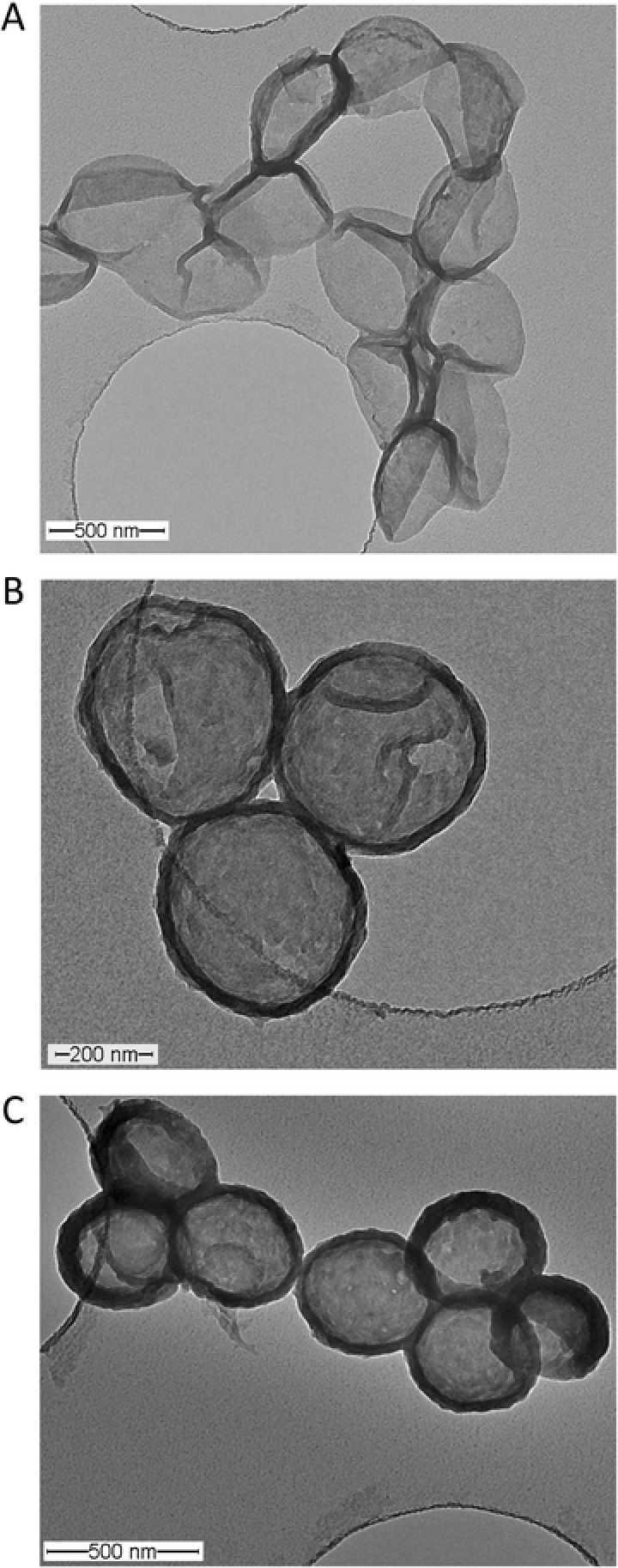
TEM images of A) [PAH/PNMB11]_2_/PAH/PSS, B) [PAH/PNMB11]_3_/PAH/PSS, and C) [PAH/PNMB11]_4_/PAH/PSS‐coated capsules, photo‐cross‐linked for 30 min (prepared using 500 nm diameter SiO_2_ particles).

To explore the minimum number of bilayers deposition in multilayers needed to keep the stability of cross‐linked hollow capsules for cargo post‐encapsulation and release, TEM analysis was performed on capsules with 2–4 PAH/PNMB11 bilayers and 1 protective PAH/PSS bilayer (**Figure** **4**). We observed that four or five bilayers deposition in total is enough to keep the spherical shape of the capsules due to high membrane thickness and stability. Moreover, additional photo‐cross‐linking of the hollow capsules consisting of [PAH/PNMB11]_4_/PAH/PSS further enhanced the membrane stability as presented in Figure 4C. Furthermore, dynamic light scattering (DLS) results obtained for five times cyclic switching between pH 5.5, 7.4, and 8.5 (Figure 4) confirmed the presence of the desired stable and responsive capsules with corresponding diameters between 490 and 550 nm in liquid environment.

Thus, the formation of stable and reversible‐responsive hollow capsules by LbL assembly after optimization of bilayer number and cross‐linking density was successfully proven by the use of zeta potential, transmission electron microscopy (TEM), scanning electron microscopy (SEM), and DLS and those capsules could now be further investigated with regard to their permeability, durability, and controllable membrane functions.

### 2.4 Temperature and pH Dual‐Responsive Hollow Capsules Characterization

**Figure 5:**
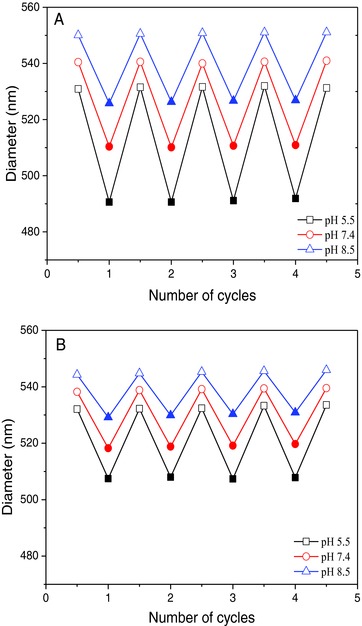
Reversible swelling‐shrinking of [PNMB11/PAH]_3_/PAH/PSS capsules photo‐cross‐linked A) 10 and B) 30 min, upon switching between 25 and 45 °C at pH 5.5, pH 7.4, and pH 8.5 buffer, respectively (open symbol = 25 °C; solid symbol = 45 °C) (*n* = 3). The maximal standard deviations of (A) and (B) are ±3.35 and ±3.04, respectively.

To confirm the temperature and pH dual‐responsive characteristics and the stability of the hollow capsules with different crosslinking degree, DLS study was used to investigate their cyclic swelling and shrinkage in aqueous solution of different pH and at temperatures between 25 and 45 °C (Scheme 1 and **Figure** **5**). In essence, the membrane of the hollow capsules, consisting of [PAH/PNMB11]_3_/PAH/PSS photo‐cross‐linked for 30 min, can undergo on/off switches for at least four cycles on stimuli, pH, and temperature. This implies that it is possible to achieve hollow capsules with tunable membrane permeability characteristics possibly suited for controlling the traffic of biomacromolecules from or into the capsule lumen depending on the respective pH and temperature stimulus. Furthermore, the permeability and durability of such photo‐cross‐linked capsules can be additionally tailored by controlling the degree of cross‐linking. For example (Figure 5A), partial cross‐linking of the same hollow capsules (10 min irradiation) resulted in a higher swelling power in different pH buffer compared to those irradiated for 30 min (Figure 5B). In addition, those capsules also showed good temperature sensitivity over four cycles of swelling and shrinking with defined changes in their diameters.

In detail, above LCST, the PNIPAM segments became hydrophobic and immediately shrink as a result of the reversible coil‐to‐globule transition (Figure 5A; 10 min photo‐cross‐linking). In contrast to the shrinking of PNIPAM segments, the deprotonated carboxylic acid units in PNMB block copolymers chains at pH 8.5 lead to electrostatic repulsion and swelling of multilayer.^61^ Therefore, the membrane of the hollow capsules CP1 and CP2 (Scheme 1 CP1 for swollen state and CP2 for shrunken state) exhibits a simultaneous competition between shrinking and swelling characteristics at different pH values and 25 °C (below LCST) and 45 °C (above LCST). Keeping the temperature at 45 °C (above LCST), the hollow capsules shrink from about 526 nm at pH 8.5 to about 490 nm at pH 5.5 due to less deprotonated acid groups and therefore a lower degree of anionic repulsion forces. This observed change of the capsules diameters impressively indicates that the hollow capsules exhibit the desired pH‐responsive characteristics.

Furthermore, below LCST, also stronger swelling characteristics at pH 8.5 is given than at pH 5.5 (Figure 5A), but in this case the difference in the maximal swelling power of the hollow capsules (capsule diameter reduced from about 550 down to 530 nm) is relatively small in comparison to that above LCST. Here both, the thermo‐sensitive PNIPAM segments and the anionic deprotonated acid‐containing segments contribute to the swelling power of the hollow capsules.

Concluding the results of the dual‐responsive characteristics for the established hollow capsules, the diameter and stability of formed capsules are controlled by the cross‐linking period and are further fine‐tuned by pH and temperature stimuli. Scheme 1 schematically summarizes the structure–activity relationships of the hollow capsules CP1 (swollen state) and CP2 (shrunken state) simultaneously tuned by pH and temperature stimuli. This provides an exciting route for the design and fabrication of artificial cell compartments with possibly versatile membrane functions.

### 2.5 Cargo Post‐Loading and Controlled Release by Hollow Capsules

We further evaluated the potential of the photo‐cross‐linked hollow capsules, based on 30 min UV irradiation, for mimicking controlled cell compartment functions like uptake, retention, and release of cargo. The hollow capsules CP1, based on [PAH/PNMB11]_3_/PAH/PSS multilayers, were used for the study. CP1, swollen at pH 7.4 and 25 °C, can be loaded with cargo to result in the state of the hollow loaded capsules CP3 in the deswollen state (Scheme 1), after separation of non‐encapsulated cargo by dialysis at pH 5.5 and 45 °C (Figure S10, Supporting Information). The resulting cargo is retained in the lumen of CP3 as long as CP3 are in the “shrunken” state. When CP3 are triggered by decreasing temperature or base addition, CP3 will switch into a (partially) “swollen” state of the hollow capsules CP3 (Scheme 1) and their cargo can be released with a controllable rate also depending on the degree of cross‐linking.

As the first step, doxorubicin hydrochloride (Dox)^62^ and rhodamine B labeled dendritic glycopolymers of different core sizes^54^ (PEI‐Mal 5 with ∅ 5 nm and PEI‐Mal 25 with ∅ 11 nm) were chosen as model cargos with different molecular weights and sizes. For that, the solution of cargos was mixed with a concentrated dispersion of the swollen hollow capsules CP1 in PBS buffer pH 7.4 at room temperature. After the penetration of CP1 by cargo reached a steady state, the non‐encapsulated cargo was removed by dialysis in phosphate buffer pH 5.5 at 45 °C. At this solution state the CP1 capsules completely switched into the loaded “shrunken” state (CP3). Of special importance is the stable level of the UV spectra recorded after 2 and 3 d of dialysis, indicating a successful separation of non‐encapsulated cargo. The loading efficiency of Dox, PEI‐Mal 5, and PEI‐Mal 25 were determined to be about 20 ± 5%, 35 ± 5%, and 40 ± 5%, respectively (Figure S10, Supporting Information), after 3 d of dialysis. Then the release of various (macro)molecules from hollow capsules was immediately started at different conditions (presented below).

**Figure 6:**
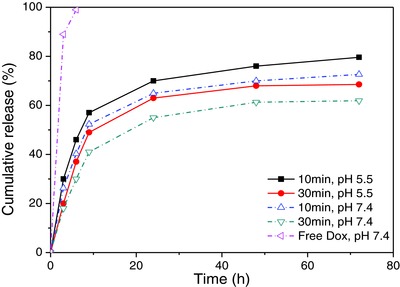
Cumulative release profile of Dox from [PNMB11/PAH]_3_/PAH/PSS capsules, cross‐linked with different time of UV irradiation and in various pH media (*n* = 3) at 45 °C. The maximal standard deviation is ±0.87.

First, Dox was used as a model of a low molecular weight drug to investigate the cross‐linking time dependence release from the hollow capsules at 45 °C (**Figure** **6**). The dialyzed Dox‐loaded capsules were immersed in solutions and analyzed using UV–vis at regular intervals as indicated in Figure 6. For different photo‐cross‐linking density of the capsules studied, Dox showed similar release profiles at physiological pH 7.4 and acidic pH 5.5, respectively. This can be described as slightly sustained release characteristic compared with free Dox. Hence, at a physiological pH 7.4, over the course of 72 h about 72% of Dox was released from cross‐linked capsules irradiated for 10 min. However, when the photo‐cross‐linking period was increased to 30 min, the high cross‐linking density can further suppress the drug diffusion from the hollow capsules. One can state in a slower release profile with a more sustained manner, in which only 61% of the Dox were released over 72 h. Thus, the permeability of the capsules and consequently the rate of drug release can be controlled by the photo‐cross‐linking period.

**Figure 7:**
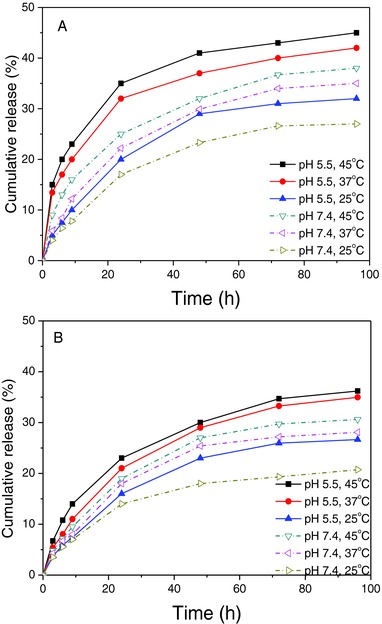
Cumulative release profile of rhodamine B labeled A) PEI‐Mal 5 and B) PEI‐Mal 25 from [PNMB11/PAH]_3_/PAH/PSS capsules, photo‐cross‐linked for 30 min, at different temperatures and in various pH medium (*n* = 3). The maximal standard deviations (A) and (B) are ±0.52 and ±0.43, respectively.

Next, the temperature and pH‐responsive release of larger nm‐sized cargos, rhodamine B‐labeled PEI‐Mal 5 (∅ 5 nm) and PEI‐Mal 25 (∅ 11 nm), from 30 min photo‐cross‐linked capsules was studied (**Figure** **7**). For this purpose, temperatures of 25 °C (below LCST), 37 °C (body temperature and slightly lower than the LCST), and 45 °C (above LCST) were selected for the release study. These temperatures were combined with both, physiological pH 7.4 and acidic environment (pH 5.5) which simulates the internal pH of cell organelles. Generally, a significantly sustained release profile for both dendritic glycopolymers is given tailored by the temperature‐ and pH‐responsive key characteristics of cargo‐containing hollow capsules CP3, as presented in Figure 7.

At physiological pH 7.4 and 25 °C, CP3 switches from “shrunken” into the “swollen” state (Scheme 1). This results in a slow release rate,^63^, ^64^ at which only 27% of PEI‐Mal 5 and 21% PEI‐Mal 25 are released within 96 h (Figure 7). In opposite to this, when the temperature is increased to 37 and 45 °C at pH 7.4 (Figure 7), the temperature‐sensitive part of the shell of the hollow capsules collapses and the capsules become smaller (Figure 5B), and this causes a slight rise of the release rate for both dendritic glycopolymers PEI‐Mal 5 and PEI‐Mal 25: over 96 h the amount of released PEI‐Mal 5 and PEI‐Mal 25 increases to 35% and 28%, respectively, at 37 °C and to 38% and 31%, respectively, at 45 °C.

On the first glance it is surprising that in the collapsed state for the capsule shell (above LCST) there is a higher release rate of the cargo macromolecules compared to that of swollen capsule shell (below LCST). One explanation can be that at the higher temperature of 45 °C, compared to 25 °C, the mobility of cargo macromolecules is increased and in addition, the ionic interactions of cationic cargo macromolecules with, at pH 7.4, anionic acid groups in the shell or membrane might be weakened. Therefore, slightly more cargo macromolecules can be released from the hollow capsules even in their collapsed state.

Besides this temperature sensitivity of the hollow capsules, the pH‐responsive characteristics of cargo‐containing hollow capsules were investigated at either *T* < LCST or *T* > LCST (Figure 7). For example, upon decreasing pH from 7.4 to 5.5 above LCST (45 °C), over 96 h the release of the entrapped PEI‐Mal 5 increases from 38% to 45%, respectively, and of PEI‐Mal 25 from 31% to 36%, respectively. This implies that the release behavior and the release characteristics of PEI‐Mal 5 and PEI‐Mal 25 from photo‐cross‐linked capsules correlate with the pH of release medium and accelerate with a pH decrease.

Considering the results from both stimuli, we can assume that electrostatic interactions between charged cargo macromolecules and the membrane of the hollow capsules are actively responding to the pH variation, and those are significantly involved in the retention capability of the capsules. As the pH drops down to pH 5.5, the carboxyl groups in the capsules shell are mainly protonated. This consequently leads to reduced electrostatic interactions between cationic cargo macromolecules and carboxyl groups in the capsules shell and results in boosting cargo macromolecules to get off the hollow capsules. Additionally, the shrinkage/swelling characteristics of the hollow capsules, mainly determined by the temperature‐sensitive part of the membrane, also have a certain effect on the cargo release (Scheme 1). With decreasing the pH and raising *T*, the capsules shrink and undoubtedly reduce their inner volume of the cavity and this goes along with a reduction in the anionic repulsion forces in the capsules shell. Thus, the cargo macromolecules are squeezed out more intensively and, finally, the release quantity of the cargo macromolecules increases.^65^

Summarizing the uptake, retention, and release of the three different cargos by the hollow capsules, we can state that for small molecules similar profiles of sustained release are obtained at 45 °C (above LCST) with a notable but weak effect of cross‐linking time and pH. For the cationic cargo macromolecules PEI‐Mal 5 (∅ 5 nm) and 25 (∅ 11 nm) the following observations can be stated: (A) a slower release of both cargo macromolecules can be found in the case of pH 7.4 and temperature below LCST (being in the “swollen” state); (B) a higher release of both cargo macromolecules is taken place in the case of pH 5.5 and temperature above LCST (being in the “shrunken” state); (C) the smaller cargo PEI‐Mal 5 reveals a slightly larger release from hollow capsules compared to the PEI‐Mal 25. Overall, a general size‐dependent permeability of the hollow capsules at each release condition is observable. This is attributed to a controlled trans‐membrane diffusion process;^66^ (D) a further sustained release of cargo can be obtained when further increasing the photo‐cross‐linking period (>30 min). Therefore, we can state that our photo‐cross‐linked hollow capsules exhibited the desired dual‐responsive behaviors usable for mimicking cell compartment functions with controllable uptake and release.

## 3 Conclusions

We have developed a novel and convenient approach for the fabrication of dual stimuli‐responsive and photo‐cross‐linked hollow capsules with tunable membrane permeability. To achieve those polymeric capsules first consecutive layer‐by‐layer deposition of cationic polyallylamine and anionic dual‐responsive photo‐cross‐linkable block copolymer PNMB was performed on planar substrate to fabricate an ultrathin multilayer for mimicking the membrane function of hollow capsules. These thin multilayers with thickness in the range of 20 nm exhibited the desired dual thermo‐ and pH‐responsive behaviors in the non‐photo‐cross‐linked and photo‐cross‐linked state. This LbL concept could be successfully transferred onto silica particles with diameters of 0.5 µm as templates. Well‐defined hollow capsules were smoothly obtained after template removal and visualized by TEM and SEM study, while their average sizes (490–530 nm in the shrunken state) were determined by DLS measurements. The uniform photo‐cross‐linked hollow capsules exhibit reversible stimuli‐responsive behavior toward changing environmental conditions, as there are pH and temperature. In addition, the capsules demonstrate high robustness and stability over several swelling/shrinkage cycles at pH 5.5, 7.4, and 8.5 combined with temperature switches between 25 and 45 °C. For mimicking cell functions with tunable membrane permeability of those hollow capsules post‐encapsulation and release of biological‐active (macro)molecules can be adapted depending on the cross‐linking density, the size of cargo, and the stimuli temperature and pH. For a low molecular weight model drug molecule doxorubicin, a high release with a low degree on retarding release profile was observed, whereas protein‐like macromolecules such as spherical dendritic glycopolymers with average diameters of 5 and 11 nm, respectively, were retarded released over 4 d up to a maximum of 45% and 36%, respectively. Thus, the dual stimuli‐responsive and photo‐cross‐linked hollow capsules have the potential for mimicking cell membrane functions with controllable diffusion process for post‐encapsulating and releasing small molecules as well as larger proteins (≤11 nm) at pH 7.4 and 37 °C and at pH 5.5 and 37 °C, respectively. This is caused by the interplay of various key characteristics such as the pH‐dependent degree of protonation of carboxylic acids in MA block, the temperature‐sensitive PNIPAM block, the non‐covalently driven interactions with hollow capsules membrane, and the cross‐linking period to induce retarding release profiles of nm‐sized (bio)macromolecules. Moreover, it cannot be excluded that some part of the protein mimics are probably entrapped in the cavity after uptake process and hollow capsules' membrane will present a natural barrier also responsible for the retarding profile of nm‐sized protein mimics under selected release conditions. Overall this approach enables us further to design and fabricate advanced and stimuli‐responsive capsules with complex architectures, such as multicompartmentalized cellular structures, for mimicking complex biological processes.

## 4 Experimental Section

*Synthesis of PNMB Block Copolymers*: PNMB block copolymer was synthesized by RAFT polymerization, as follows: (1) Synthetic step for various macro‐CTA: RAFT agent 2‐(dodecylthiocarbonothioylthio)‐2‐methylpropionic acid (0.1 mmol, 36.5 mg), AIBN (0.01 mmol, 1.64 mg), *N*‐isopropyl acrylamide (6.67 mmol for PNMB21, 5 mmol for PNMB11, 3.33 mmol for PNMB12, or 2 mmol for PNMB14, respectively) were added to a 10 mL round‐bottom flask and degassed for 30 min. Then 1,4‐dioxane (3 mL) was deoxygenated in a second separated flask by purging nitrogen for 15 min and then solvent was transferred to the reaction flask under protection atmosphere. The mixture was stirred at 60 °C for 12 h. To quench the polymerization solution, a rapid cooling of the reaction flask in liquid nitrogen was carried out. The 1,4‐dioxane‐containing monomer/polymer mixture was twice poured into diethylether to precipitate the PNIPAM macro‐CTA, followed by the removal of the PNIPAM macro‐CTA by filtration and, finally, dried under suction. Yield: 75%. The dispersity (*Ð*) of the various PNIPAM macro‐CTAs was 1.10 for PNMB21, 1.08 for PNMB11, 1.05 for PNMB12, and 1.05 for PNMB14, respectively. Thus, different PNIPAM macro‐CTAs were established for the synthesis of PNMB block copolymers (Table 1). (2) Synthetic step for various block copolymers: PNIPAM macro‐CTA (0.1 mmol), AIBN (0.01 mmol, 1.64 mg), and the corresponding amount of the cross‐linker monomer BMA (2 mmol, 846.9 mg) were added to a 10 mL round‐bottom flask and degassed for 30 min. Then 1,4‐dioxane (6 mL) and methacrylic acid (3.33 mmol for PNMB21, 5 mmol for PNMB11, 7.33 mmol for PNMB12, or 8.6 mmol for PNMB14, respectively) were deoxygenated in a second separated reaction flask by purging nitrogen for 15 min before transferred to the reaction flask under protection atmosphere. The mixture was stirred at 60 °C for 2 h. To quench the polymerization solution, a rapid cooling of the polymerization solution in liquid nitrogen was carried out. The 1,4‐dioxane‐containing monomer/polymer mixture was twice poured in diethylether to precipitate the desired PNMB block copolymer, followed by the removal of the block copolymer by filtration and dried under suction. The conversion rate of MA and BMA in the PNMB block copolymers was 75% for PNMB21, 74% for PNMB11, 72% for PNMB12, and 70% for PNMB14, respectively.

*Preparation of Hollow Capsules by Using LbL Assembly*: Multilayers of PAH and PNMB11 were adsorbed on silica particles with diameters of 500 nm using the LbL method. The corresponding polyelectrolyte solutions for PAH, PNMB11, and PSS were prepared, using a concentration of 1 mg mL^−1^ in 0.4 m NaCl. Then, the pH of the solutions was adjusted to 7.5. All solutions were filtered through a 0.2 µm nylon filter prior to use. The initial 100 µL suspension of 500 nm diameter silica particles were centrifuged (6000 g for 2 min) and the supernatant was removed. For the initial washing step, MilliQ water was added, the particles redispersed and centrifuged again. This procedure was repeated three times before multilayers' formation on silica particles could be started. Subsequently, PAH was added to the freshly washed and bare silica particles and allowed to adsorb for 30 min with constant shaking (to deposit the first layer of the multilayered shell). The particles were then worked up by three centrifugation/dispersion cycles with MilliQ water. PNMB11 was then adsorbed to the particles for 30 min, which were subsequently washed. This procedure was then repeated to deposit the desired bilayers as mentioned in the main text. After washing three times in MilliQ water, PAH and PSS were then adsorbed to the particles to form the protective capping bilayers.

*Photo‐Cross‐Linking of the Multilayers on Silica Particles*: The solution of particles was sonicated for 30 min before UV light cross‐linking started. Then the solution was placed in the UV chamber equipped with a low intensity (0.1 W cm^−2^) iron lamp and irradiated for the desired period (5, 10, 20, 30, or 50 min). The capsules were left overnight prior to removing the silica core template.

*Preparation of the Hollow Capsules via Silica Core Template Removal*: The particle suspensions were mixed well by vortexing and transferred to Eppendorf tubes. The particles were worked up by one centrifugation/dispersion cycles with MilliQ water (100 µL). The template silica cores were then removed to fabricate hollow capsules by the addition of a hydrogen fluoride (HF) buffered to pH 7.3 with ammonium fluoride (NH_4_F). *(Caution! Note that hydrogen fluoride and ammonium fluoride are highly toxic. Extreme care should be taken when handling HF solution and only small quantities should be prepared.)* The samples were tapped gently to dissolve the silica cores for about 30 min. The excess NH_4_F, HF, and SiF_4_ were removed from hollow capsules by three centrifugation/dispersion cycles with MilliQ water, followed by re‐suspending in a suitable volume (typically 0.5 mL) of MilliQ water.

## Supporting information

As a service to our authors and readers, this journal provides supporting information supplied by the authors. Such materials are peer reviewed and may be re‐organized for online delivery, but are not copy‐edited or typeset. Technical support issues arising from supporting information (other than missing files) should be addressed to the authors.

Supplementary
