# Supplementary material for: Photo‐Cross‐Linked Dual‐Responsive Hollow Capsules Mimicking Cell Membrane for Controllable Cargo Post‐Encapsulation and Release
Source: Adv Sci (Weinh). 2016 Dec 12;4(3):1600308. doi: 10.1002/advs.201600308 (PMC5357983; doi:10.1002/advs.201600308)
Supplement: Supplementary file 1 — Supplementary [file ADVS-4-na-s001.pdf]

Copyright WILEY-VCH Verlag GmbH & Co. KGaA, 69469 Weinheim, Germany, 2016.

ADVANCED SCIENCE
Open Access

Supporting Information

for *Adv. Sci.,* DOI: 10.1002/advs.201600308

Photo-Cross-Linked Dual-Responsive Hollow Capsules Mimicking Cell Membrane for Controllable Cargo Post-Encapsulation and Release

Xiaoling Liu, Dietmar Appelhans, Qiang Wei, and Brigitte Voit**

Copyright WILEY-VCH Verlag GmbH & Co. KGaA, 69469 Weinheim, Germany, 2013.

Supporting Information

Photo-crosslinked Dual-responsive Hollow Capsules Mimicking Cell Membrane for Controllable Cargo Post-encapsulation and Release

Xiaoling Liu, Dietmar Appelhans, Qiang Wei, Brigitte Voit,**

X. Liu, Dr. D. Appelhans, Q. Wei. Prof. B. Voit
Leibniz-Institut für Polymerforschung Dresden e.V., Hohe Straße 6, D-01069 Dresden, Germany
E-Mail: applhans@ipfdd.de, voit@ipfdd.de

X. Liu, Q. Wie, Prof. B. Voit
Organic Chemistry of Polymers, Technische Universität Dresden, D-01062 Dresden, Germany

Table of Contents for Supporting Information:

1. EXPERIMENTAL SECTION 4

1.1 Materials 4

1.2 Characterization methods 5

1.3 Synthesis of PRMNB random copolymer 7

1.4 Preparation of Multilayers Assembly on Planar Substrates 7

1.5 Reversible swelling-shrinking behavior of the crosslinked multilayer assembly films on planar substrates 8

1.6 Reversible swelling-shrinking behavior of the crosslinked hollow capsules 9

1.7 Encapsulation of doxorubicin 9

1.8 *In vitro* release studies for Dox 9

1.9 Rhodamine B labeled maltose-decorated hyperbranched poly(ethylene imine) macromolecules (PEI-Mal 5 and PEI-Mal 25) 10

2.0 Encapsulation of PEI-Mal 5 and PEI-Mal 25 11

2.1 *In vitro* release studies for PEI-Mal 5 and PEI-Mal 25 11

2.2 Turbidity study on block and random copolymers 12

2. SUPPORTING FIGURES 13

Scheme S1. Schematic illustration of the fabrication of photo-crosslinked hollow capsules. 13

Figure S1. ^{1}H-NMR spectrum (DMSO-d6) of PNMB11 with all peaks labeled to the corresponding groups in the molecule. ^{1}H NMR signals of NIPAM at 3.84 ppm, BMA at 6.3-7.7 ppm, and MA at 12-12.75 ppm, respectively. 14

Figure S2. A) Transmittance curve upon heating for LCST measurements of block copolymer PNMB21, PNMB11, PNMB12, and PNMB14 at pH 7.5 (UV-vis spectroscopy). B) Reversible volume phase transition of PNMB11 upon switching between 25 ℃ and 45 ℃ at pH 7. (open symbol=25 ℃; solid symbol=45 ℃). 15

Figure S3. Heating curves of the LCST measurements of random polymer PRMNB, block copolymer PNMB11 and homopolymer PNIPAM at pH 7.5, followed by transmittance (UV spectroscopy). 16

Figure S4. Heating curves of the LCST measurements of block copolymer PNMB11 at pH 5.5, pH 6.5, pH 7.5, pH 8.5 and pH 9.5, respectively, followed by transmittance (UV-vis spectroscopy). 17

Figure S5. Zeta potential data of A) PAH and B) PNMB11 as a function of pH. 18

Figure S6. Reversible swelling-shrinking of multilayer films [PAH/PNMB11]$_3$/PAH/PSS on planar substrate after photo-crosslinking for 10 min (A) and 20 min (B) upon switching between temperature 25℃ and 45℃ at pH 5.5, pH 7.4 and pH 8.5 buffer, respectively (open symbol=25 ℃; solid symbol=45 ℃) (n=3). 18

Table S1. Swelling ratio of multilayer films [PAH/PNMB11]$_3$/PAH/PSS on planar substrate at different pH values below and above LCST. 19

Figure S7. TEM images of [PAH/PNMB11]3/PAH/PSS-coated capsules, photo-crosslinked for 0 min (A), 5 min (B), 10 min (C), 20 min (D), 30 min (E) and 50 min (F), respectively. 20

Figure S8. Schematic overview and UV-Vis monitoring how cargo is loaded into photo-crosslinked [PNMB11/PAH]$_3$/PAH/PSS hollow capsules. Capsule membrane allows for transmembrane diffusion at pH 7.4 and 25 ℃. When cleaned afterwards at pH 5.5 and 45 ℃ using dialysis (dialysis tube MWCO 5000 for Dox and MWCO 150 kDa for PEI-Mal 5 and PEI-Mal 25, respectively), the Dox signal (bottom, left), rhodamine B labeled PEI-Mal 5 (bottom, middle) and PEI-Mal 25 (bottom, right) were still detectable and remained at a stable level, indicating a successful enclosure. 21

Figure S9. Calibration curves of Dox in phosphate buffer at pH 5.5 and in PBS at pH 7.4. The measurement was carried out at 488 nm using UV-vis spectrophotometer. 22

Figure S10. Calibration curves of the rhodamine B labeled PEI-Mal 5 (A) and PEI-Mal 25 (B) in phosphate buffer pH 5.5 and PBS pH 7.4. The measurement was carried out at 500 nm using UV-vis spectrophotometer. 22

3. REFERENCE 23

1. EXPERIMENTAL SECTION

1.1 Materials

All reagents and solvents were purchased from commercial suppliers and used as received unless otherwise noted. 2-(dodecylthiocarbonothioylthio)-2-methylpropionic acid (98 %), 2,2′-azobis(2-methylpropionitrile) (AIBN, 98 %), 1,4-dioxane (anhydrous, 99.8%), hydrofluoric acid (HF, 48%), ammonium fluoride (NH_4F, ≥98.0%), poly(sodium 4-styrenesulfonate) (PSS, molecular weight 70,000), sodium chloride (NaCl, ≥99.5%), ammonia solution (NH_4OH, 29 wt.-% aqueous solution), hydrogen peroxide (H_2O_2, 30 wt.-% aqueous solution), aluminum oxide (neutral, activated), D-maltose monohydrate (Mal) and rhodamine B isothiocyanate, phosphate buffered saline (tablet), monosodium phosphate and disodium phosphate were purchased from Sigma-Aldrich. 2-hydroxy-4-(methacryloyloxy) benzophenone (BMA, 99%) and Poly(allylamine hydrochloride) (PAH, molecular weight 70,000 g mol^{-1}) were purchased from Alfa Aesar. Hyperbranched poly(ethylene imine) (PEI 5, M_w= 5000 g mol^{-1}) and hyperbranched poly(ethylene imine) (PEI 25, M_w= 25000 g mol^{-1}) were obtained from BASF SE (Ludwigshafen, Germany). Doxorubicin hydrochloride (Dox, 98.0-102.0%) was purchased from Carbosynth Limited. 500nm diameter SiO_2 particles (5 wt.-%) were obtained from Microparticles GmbH, Germany. Methacrylic acid (MA, Sigma, 99%) was passed through an inhibitor removing column (neutral aluminum oxide) prior to being stored under an argon atmosphere in the refrigerator. The monomer *N*-isopropyl acrylamide (NIPAM, Sigma, 97%) was purified by recrystallization, from an *n*-hexane/toluene mixture (90/10 vol.-%). Dialysis tubes made from regenerated cellulose (MWCO 5000 and MWCO 150 kDa) were purchased from Carl Roth. High purity and resistivity

(> 18 M Ω cm) deionized water (MilliQ water) was obtained from an inline Milli-Q Reagent Water Purification System (Millipore Corporation) and was used in all the reactions, solution preparations, and polymer isolations.

1.2 Characterization methods

Gel Permeation Chromatography. The molecular weight distributions of the copolymers were measured at 40 ℃ using a Polymer Laboratories PL-GPC50 Plus Integrated GPC system(Varian Inc., UK) equipped with a Polymer Laboratories pump, a PL ResiPore column (300 × 7.5 mm), a PL data stream refractive index detector, and a PL-AS-RT auto sampler. The calibration was carried out using 12 polystyrene standards with M_n values ranging from 162 to 371,100 g mol^{-1} (Varian Inc., UK). The eluent was DMAc, and the flow rate was 1.0 mL min^{-1}. The data were processed using Cirrus GPC offline GPC/SEC software (version 2.0).

NMR spectroscopy. ^1H NMR spectra were recorded using Bruker Avance III 500 spectrometer operating at 500.13 MHz and DMSO-d6 as solvent at room temperature. Molar masses of the block copolymers, lengths of their block and the ratio of MA and NIPAM and the percent of BMA incorporated in the thermo-sensitive block were determined from ^1H NMR spectra. In detail, every characteristic hydrogen signal of the three monomers, MA, NIPAM and BMA, can be found in the polymer chain, indicating copolymerization took place. Furthermore, the ratio of MA and NIPAM in the copolymer chain was calculated by the integration of the ratio between the specific signals of MA at 12-12.75 ppm and NIPAM at 3.84 ppm, respectively, and the content of PBMA was calculated to be about 5% by the integration of the ratio between the specific signals of BMA (6.3-7.7 ppm) and PNIPAM (3.84 ppm), respectively.

UV/vis spectroscopy. The lower critical solution temperature (LCST) of the polymers was measured on a Tepper TP1 photometer (Mainz, Germany). Transmittance of the polymer in

MilliQ water at 670 nm was monitored as a function of temperature (cell path length: 12 mm; one heating/cooling cycle at a rate of 1 °C min^{-1}) and the critical temperature was determined at 50% of relative transmittance.

Zeta potential. Zeta potential of the polyelectrolytes PAH and PNMB11 was determined using Zetasizer Nano-series instrument (Malvern Instruments, UK) equipped with a multi-purpose autotitrator. PAH (1 mg mL^{-1}) without salt (pH adjusted with HCl to pH 4) was titrated with NaOH solution and PNMB (1 mg mL^{-1}) without salt (pH adjusted with NaOH to pH 9.5) was titrated with HCl solution.

Zeta potential values of the bare and multilayered silica particles were measured on a ZETASIZER Nano series instrument (Malvern Instruments, UK) with a Dispersion Technology Software (version 7.10). The data was collected by three repeated experiments at 25 ℃ with a voltage of 150V.

Photo-crosslinking of multilayers. UV irradiation of polymer (multi)layers was performed using UVACUBE100 (honle UV Technologies, Germany) equipped with a low intensity (0.1 Wcm^{-2}) iron lamp as UV source.

Ellipsometry. The film thicknesses measurements were performed on a UVISEL spectroscopic ellipsometer (HORIBA Jobin Yvon S.A.S, Chilly Mazarin, France). Spectroscopic data was acquired between 400 and 800 nm with a 2 nm increment, and unless otherwise stated, thicknesses were extracted with the integrated software by fitting with a classical wavelength dispersion model. No less than three measurements were taken on each sample.

Dynamic light scattering. The hydrodynamic size of capsules were determined by dynamic light scattering (DLS) using Zetasizer Nano-series instrument (Malvern Instruments, UK)

equipped with a 633 nm He-Ne laser at fixed scattering angle of 173°. The data were analyzed using software version 6.12.

Transmission electron microscopy. The diameters and morphologies of the capsules were observed using a transmission electron microscopy (TEM) Libra 120 equipped with a charge coupled device (CCD) camera at an accelerating voltage of 120 kV. 5 μ L of the capsules or particles were dispersed in water with the concentration of 1 mg mL^{-1} and allowed to adsorb for 5 min. onto a 300 mesh, carbon film coated copper grid, and the specimen was dried at room temperature or the grid was blotted dry using filter paper.

SEM samples were prepared from 1 μL of a concentrated capsule solution on Si wafers which were sputter-coated with gold and were analyzed using a Zeiss Ultra 55 Gemini scanning electron microscope.

1.3 Synthesis of PRMNB random copolymer

PRMNB random copolymer was synthesized by RAFT polymerization, as follows: RAFT agent 2-(dodecylthiocarbonothioylthio)-2-methylpropionic acid (0.1 mmol, 36.5 mg), AIBN (0.01 mmol, 1.64 mg), N-isopropyl acrylamide (5 mmol) were added to a 10 mL round-bottom flask and degassed for 30 min. Then 1,4-dioxane (5 ml) and methacrylic acid (5 mmol) were deoxygenated in a second separated reaction flask by purging nitrogen for 15 min before transferred to the reaction flask under protection atmosphere. The mixture was stirred at 60 ℃ for 15h. To quench the polymerization solution, a rapid cooling of the polymerization solution in liquid nitrogen was carried out. The 1,4-dioxane-containing monomer/polymer mixture was twice poured in diethylether to precipitate the desired PRMNB random copolymer, followed by the removal of the random copolymer by filtration and dried under suction. Yield: 75 %.

1.4 Preparation of Multilayers Assembly on Planar Substrates

Multilayers were deposited on Si wafer substrate by using LbL technique. The polyelectrolytes PAH and PNMB11 were prepared at a concentration of 1 mg mL^{-1} in 0.4 M NaCl and the pH of the solutions was adjusted to 6.0, 6.5, 7.0, 7.5, 8.0, 8.5, 9.0, 9.5 and 10.0, respectively. The solutions were filtered through a 0.2 μm nylon filter prior to use. Si wafers were first cleaned by ultra sonication in 2-propanol (100 mL) for 15 min and then hydrophilized by immersion in a 5:1:1 mixture of MilliQ water, ammonia solution NH_4OH (29 wt.-% aqueous solution), hydrogen peroxide H_2O_2 (30 wt.-% aqueous solution) at 70 ℃ for 30 min, followed by rinsing three times with MilliQ water and drying with a gentle stream of nitrogen. First, the cleaned Si wafers were dipped in PAH for 30 min to establish a precursor layer on the surface. The Si wafers were then washed three times with MilliQ water and dried with a gentle stream of nitrogen. Then, they were immersed in the anionic PNMB11 solution for 30 min, followed by further washing and drying steps. This procedure was repeated alternating between PAH and PNMB11 deposition until the desired number of bilayers as mentioned in the result part were deposited. Prior to examination, the multilayers were dried with a nitrogen flow. For studying "reversible swelling-shrinking behavior" of samples multilayers with a protective capping layers were prepared by 3 bilayers of PAH and PNMB11 and the capping bilayers of PAH and PSS.

1.5 Reversible swelling-shrinking behavior of the crosslinked multilayer assembly films on planar substrates

The polymer-coated Si wafers with different crosslinking time were incubated with phosphate buffer with different pH values 5.5, 7.4, and 8.5, respectively. At each pH state, polymer-coated Si wafers were allowed to stand for 30 min. Then the environmental temperature for the Si wafers was switched between 25 °C and 45 °C and the film thickness was determined by ellipsometric

measurements. This process was repeated for several cycles as shown in the main text. No less than 20 measurements were taken on each temperature.

1.6 Reversible swelling-shrinking behavior of the crosslinked hollow capsules

The photo-crosslinked hollow capsules prepared by different crosslinking period were incubated with phosphate buffer possessing pH 5.5 and 7.4, respectively. At each pH state, hollow capsules were allowed to stand for 20 min. Then the temperature of the capsules was switched between 25 ℃ and 45 ℃ and the particles diameters were determined by DLS. This process was repeated for several cycles as shown in the main text. No less than 20 measurements were taken on each temperature.

1.7 Encapsulation of doxorubicin

To a 5 mL doxorubicin solution (c = 0.1 mg mL^{-1}) a concentrated solution containing crosslinked capsules with 5 mol-% crosslinker was added. The mixture was shielded from light and stirred for 12h in PBS buffer pH 7.4 at room temperature. The non-encapsulated doxorubicin was removed by dialysis against a membrane with MWCO = 5000 for 3 days in 0.01 M phosphate buffer pH 5.5 at 45 ℃ (3 times a day, a standard procedure for dialysis). The same procedure was carried out with pure water instead of the capsule solution for a control experiment. After selected time points, the samples were taken and analyzed using UV-vis spectroscopy (488 nm) according to the Dox calibration curve to confirm the successful removal of the free DOX. The experiments were carried out in triplicate. The loading efficiency was calculated from the following equations:

$$loading\ efficiency\ of\ Dox = \frac{loaded\ amount\ of\ Dox}{initial\ amount\ of\ Dox} * 100$$

1.8 *In vitro* release studies for Dox

As a general rule, all experiments were carried out in triplicate and the average values were plotted. For the release behavior of capsules, the Dox-loaded capsules were immersed in solutions to study the effects of pH and cross-linking density on release kinetics. Thus, 0.01 M PBS pH 7.4 and 0.01 M phosphate buffer pH 5.5 were used as release media. Dox loaded capsules solution (5 ml) was transferred to dialysis tube (MWCO 5000). The dialysis tube was sealed and then allowed to stir (200 rpm) in beaker containing 2 liter of the corresponding release medium at 45 ℃. At selected interval times, samples were removed from the dialysis tubes and quickly analyzed by UV–Vis spectroscopy (488 nm) and returned back into the dialysis tube. The amount of drug released was calculated from the amount of drug initially present in the capsules and the amount of drug retained in the capsules at each sampling point.

1.9 Rhodamine B labeled maltose-decorated hyperbranched poly(ethylene imine) macromolecules (PEI-Mal 5 and PEI-Mal 25)

For the encapsulation experiments with PEI-Mal 5 and PEI-Mal 25 in this study, we have used positively charged maltose-decorated poly(ethylene imine) with PEI core of 5000 Da (PEI-Mal 5) and 25000 Da (PEI-Mal 25). PEI-Mal 5 and PEI-Mal 25 possess an open maltose shell which is finally described as structure B by Appelhans et al.[1] PEI-Mal 5 (∅ 5 nm) and PEI-Mal 25 (∅ 11 nm) were synthesized and characterized as previously described in reference.[1] To detect PEI-Mal nanoparticles in the following experiments, they were labeled with a UV-vis active dye rhodamine B. We labeled these molecules as follows: rhodamine B (1.0 mg) was dissolved in DMSO (0.2 ml). Then, PEI-Mal 5 or PEI-Mal 25 (50 mg), respectively, was dissolved in deionized water (1 mL). Both solutions were mixed and stirred overnight. Non-bound rhodamine B was removed by dialysis method. Powders of the materials were obtained by freeze drying process.

2.0 Encapsulation of PEI-Mal 5 and PEI-Mal 25

A concentrated solution containing the capsules with 5 mol-% crosslinker and photo-crosslinked for 30 min. was added to a PEI-Mal 5 (c = 2 mg mL^{-1}) and PEI-Mal 25 solution(c = 6 mg mL^{-1}), respectively. The mixture was shielded from light and stirred for 12h in PBS buffer pH 7.4 at room temperature. The non-encapsulated PEI-Mal is removed by dialysis against a membrane with MWCO = 150 kDa for 3 days in phosphate buffer pH 5.5 at 45 °C (3 times a day, a standard procedure for dialysis). After the time points, the samples were taken and analyzed using UV-vis (500 nm) according to the calibration curves of rhodamine B labeled PEI-Mal 5 and PEI-Mal 25 in phosphate buffer at pH 5.5 to confirm the successful removal of the free rhodamine B. The experiments were carried out in triplicate. The loading efficiency was calculated from the following equations:

$$loading\ efficiency\ of\ PEI\text{-}Mal = \frac{loaded\ amount\ of\ PEI\text{-}Mal}{initial\ amount\ of\ PEI\text{-}Mal} * 100$$

2.1 *In vitro* release studies for PEI-Mal 5 and PEI-Mal 25

As a general rule, all experiments were carried out in triplicate and the average values were plotted. For the release behavior of capsules, the PEI-Mal loaded capsules were immersed in solutions to study the effects of pH and temperature on release kinetics. Thus, 0.01 M PBS at pH 7.4 and 0.01 M phosphate buffer at pH 5.5 were used as release media, respectively. PEI-Mal loaded capsules solution (5 ml) was transferred to dialysis tube (MWCO 150 kDa). The dialysis tube was sealed and then allowed to stir (200 rpm) in beaker containing 2 liter of the corresponding release medium at 45 °C, 37 °C and 25 °C, respectively. At selected interval times, samples were removed from the dialysis tubes and quickly analyzed by UV-vis spectroscopy (500 nm) and returned back into the dialysis tube. The amount of PEI-Mal macromolecules

released was calculated from the amount of PEI-Mal macromolecules initially present in the capsules and the amount of PEI-Mal macromolecules retained in the capsules at each sampling point. Additionally, no change of hollow capsules´ diameter was observed during the release experiments (data not shown).

2.2 Turbidity study on block and random copolymers

The 1:1 block copolymer PNMB11 possessed a good thermo-sensitivity as found for PNIPAM homopolymers over a broad pH range up to pH 8.5 (above pH 9 the aggregated block copolymer was highly swollen owing to ionization of carboxyl groups **(Figure S4)**). However, the cloud point of the respective 1:1 random copolymer PRMNB disappeared already at pH 7.5 (**Figure S3** in the Supporting Information), because the MA components (which are ionized at pH 7.5) convey sufficient solubility to offset the aggregation of hydrophobic temperature-sensitive components. To investigate the effect of MA content on the LCST of the block copolymers, turbidity measurements were performed **(Figure S2A)**. It was observed that the LCST values of block copolymer rapidly rose with the molar fraction of MA in the block copolymer, with the cloud point increasing from 39.9 ℃ for the 2: 1 ratio of NIPAM and MA in PNMB21 to 49.0 ℃ for the 1: 4 ratio of NIPAM and MA in PNMB14 due to the hydrophilic character of the MA monomers in the polymeric block of MA and BMA. Increasing the MA content in PNMB (Figure S2A), the degree of hydrogen-bond-driven interactions in PNMB solution is higher than in pure PNIPAM homopolymer solution. Thus, there is a need for more energy to destroy the hydrogen-bond interactions which causes an increase in their LCST behavior.[2, 3]

2. SUPPORTING FIGURES

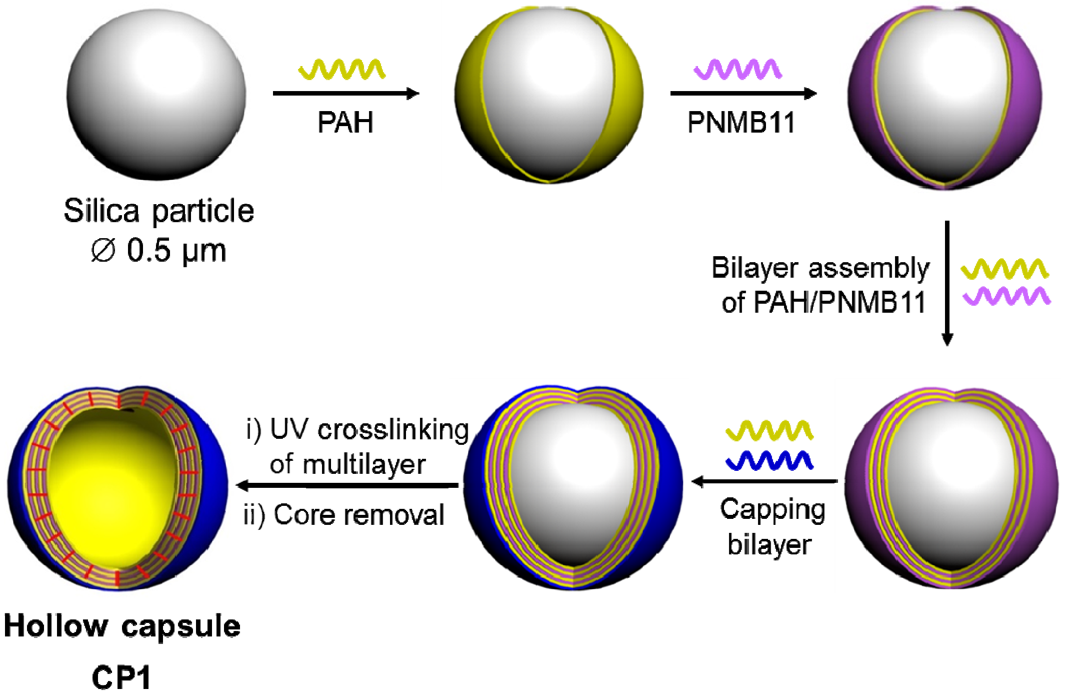

Scheme S1. Schematic illustration of the fabrication of photo-crosslinked hollow capsules.

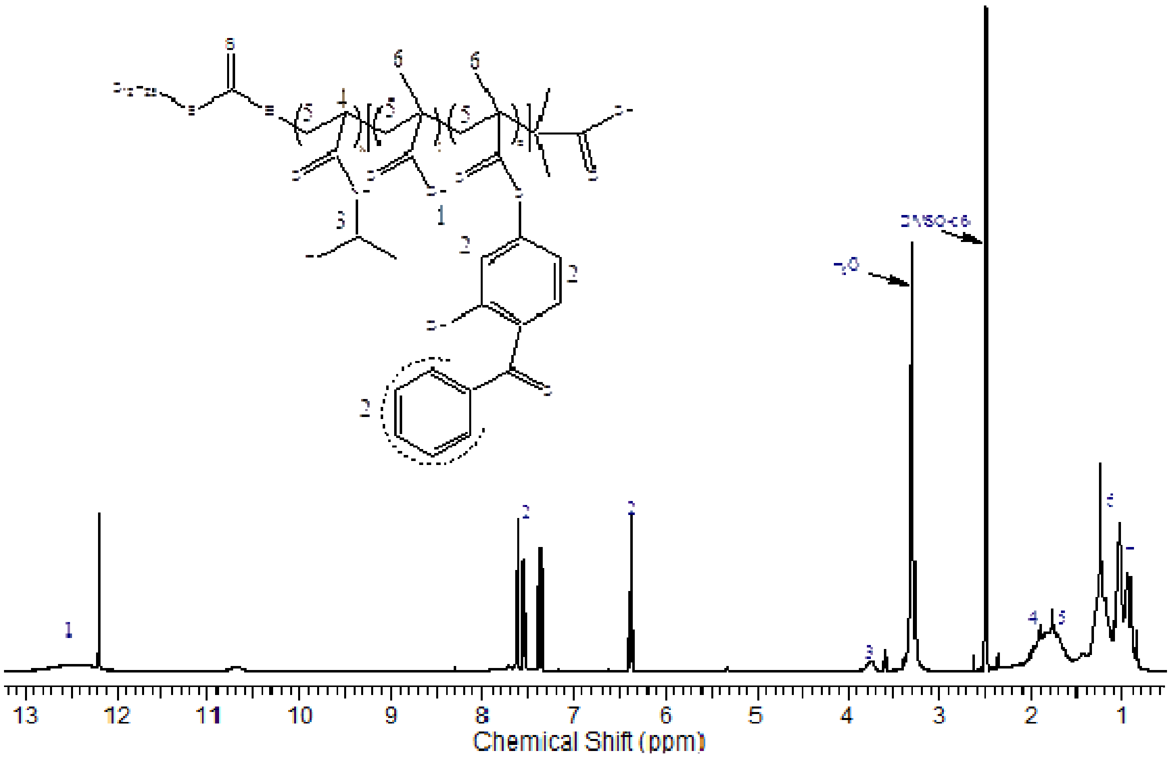

Figure S1. ^{1}H-NMR spectrum (DMSO-d6) of PNMB11 with all peaks labeled to the corresponding groups in the molecule. ^{1}H NMR signals of NIPAM at 3.84 ppm, BMA at 6.3-7.7 ppm, and MA at 12-12.75 ppm, respectively.

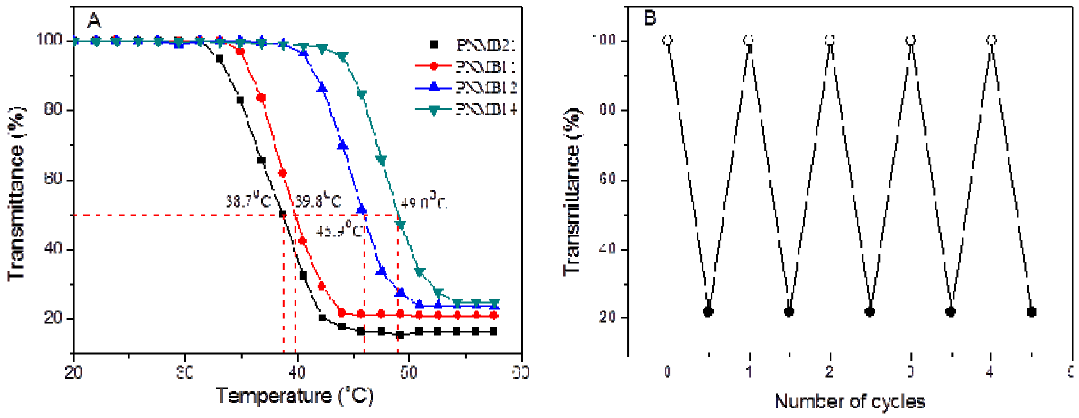

Figure S2. A) Transmittance curve upon heating for LCST measurements of block copolymer PNMB21, PNMB11, PNMB12, and PNMB14 at pH 7.5 (UV-vis spectroscopy). B) Reversible volume phase transition of PNMB11 upon switching between 25 ℃ and 45 ℃ at pH 7. (open symbol=25 ℃; solid symbol=45 ℃).

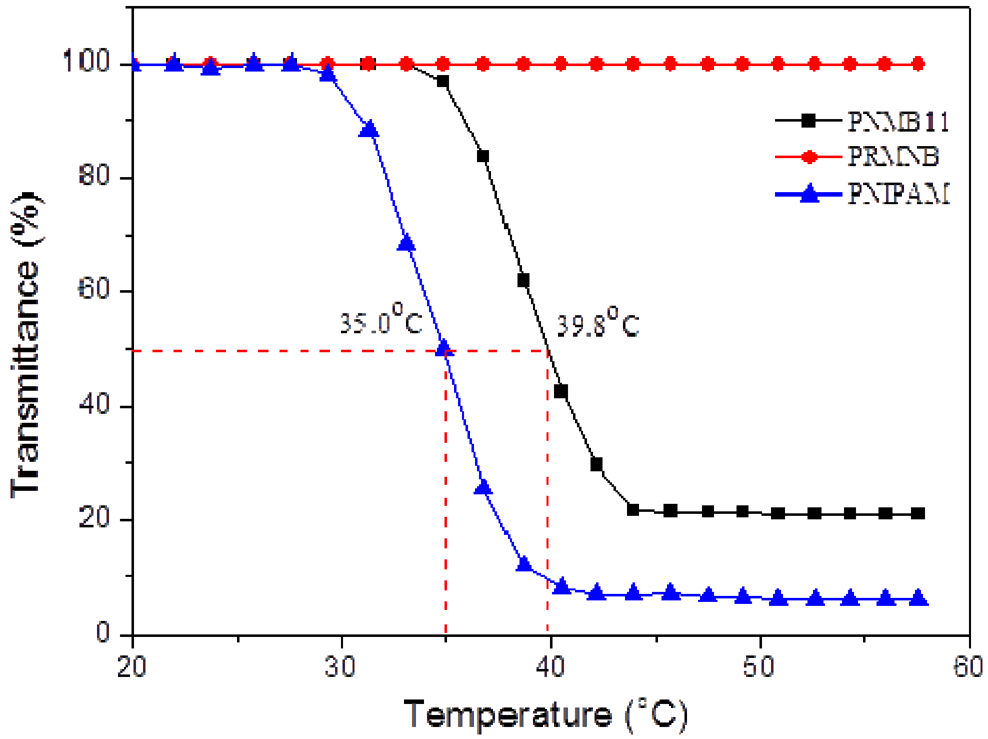

Figure S3. Heating curves of the LCST measurements of random polymer PRMNB, block copolymer PNMB11 and homopolymer PNIPAM at pH 7.5, followed by transmittance (UV spectroscopy).

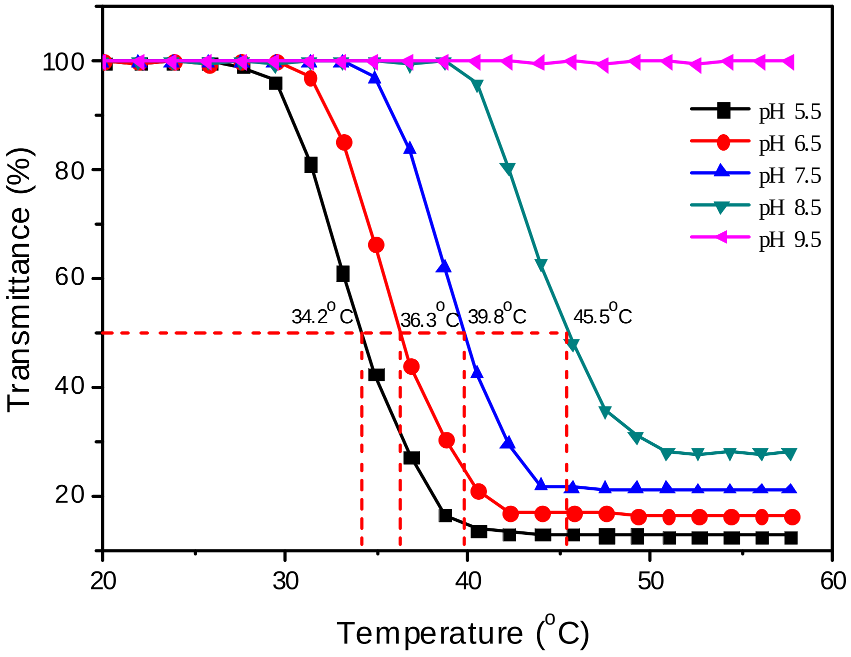

Figure S4. Heating curves of the LCST measurements of block copolymer PNMB11 at pH 5.5, pH 6.5, pH 7.5, pH 8.5 and pH 9.5, respectively, followed by transmittance (UV-vis spectroscopy).

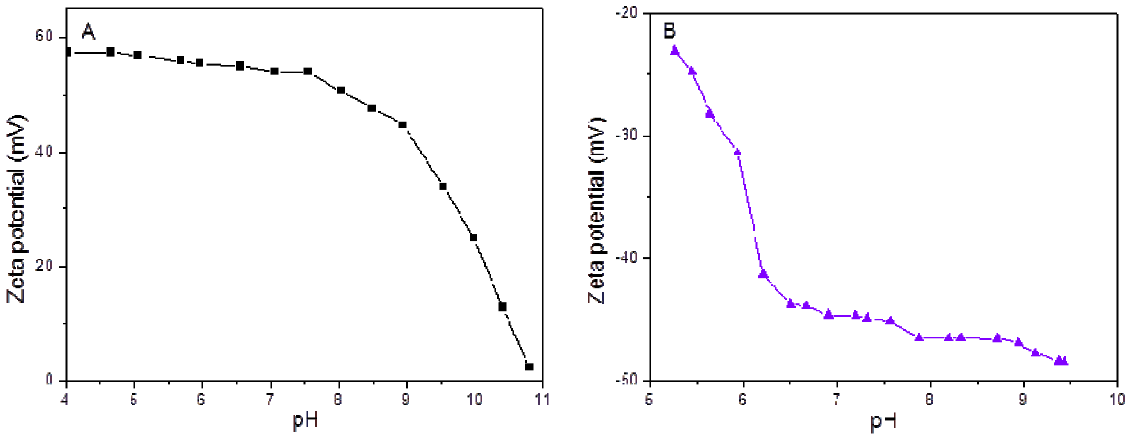

Figure S5. Zeta potential data of A) PAH and B) PNMB11 as a function of pH.

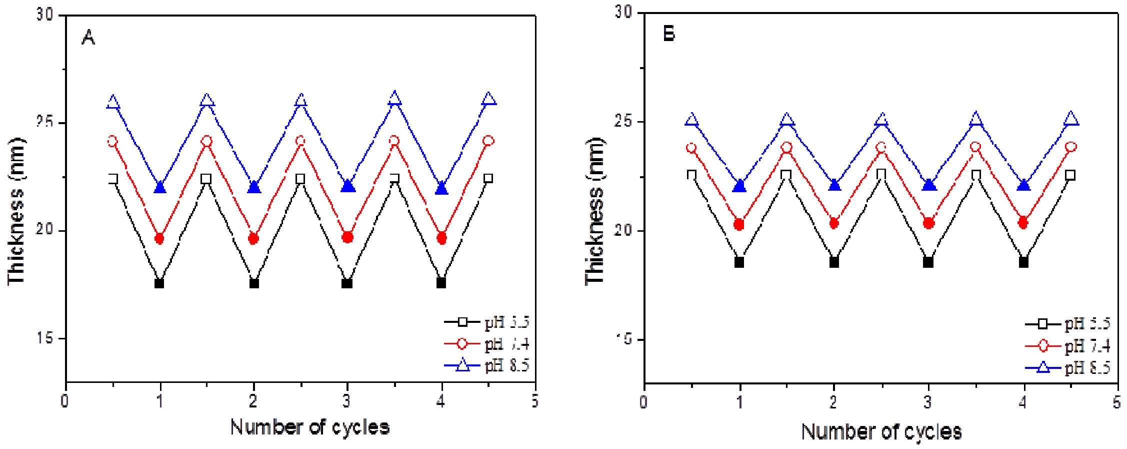

Figure S6. Reversible swelling-shrinking of multilayer films $[PAH/PNMB11]_3/PAH/PSS$ on planar substrate after photo-crosslinking for 10 min (A) and 20 min (B) upon switching between temperature 25 ℃ and 45 ℃ at pH 5.5, pH 7.4 and pH 8.5 buffer, respectively (open

symbol=25 ℃; solid symbol=45 ℃) (n=3). The maximal standard deviations of Figure S6A and S6B are ±0.56 and ±0.51, respectively.

Table S1. Swelling ratio of multilayer films [PAH/PNMB11]$_3$/PAH/PSS on planar substrate at different pH values below and above LCST

Photo-crosslinking time of multilayer films [min]	Swelling ratio between below (25 ℃) and above LCST (45 ℃)[a]		Swelling ratio between pH 8.5 and pH 5.5 [b]	
	pH 5.5	pH 8.5	25 ℃ (below LCST)	45 ℃ (above LCST)
0	1.452	1.234	1.206	1.419
30	1.185	1.089	1.087	1.182

[a], [b] Calculated from Figure 2B and 2C.

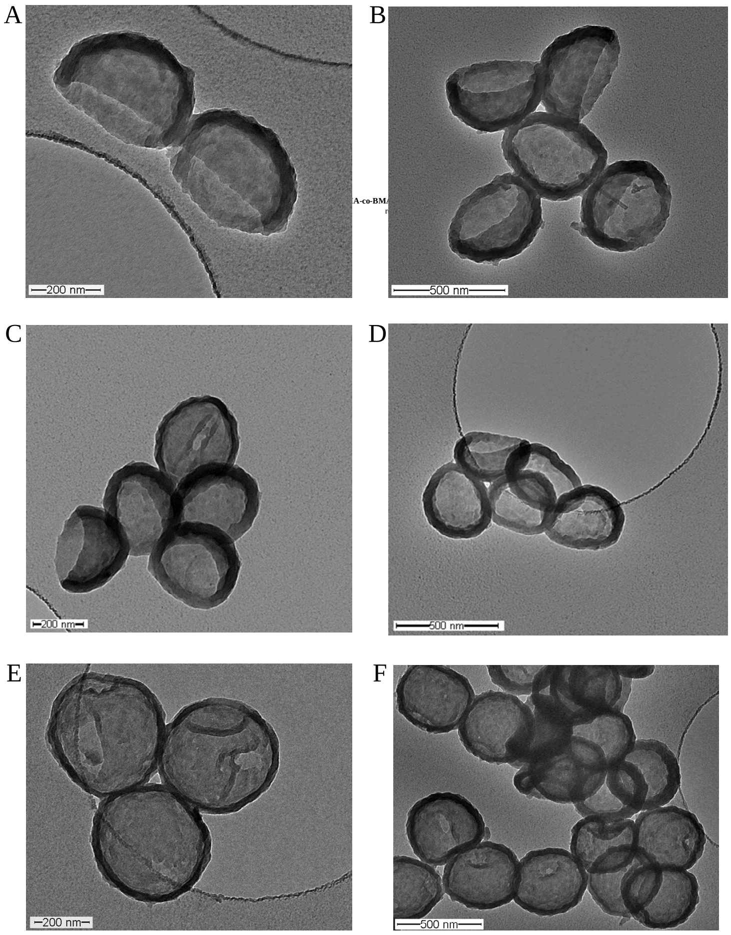

Figure S7. TEM images of [PAH/PNMB11]3/PAH/PSS-coated capsules, photo-crosslinked for 0 min (A), 5 min (B), 10 min (C), 20 min (D), 30 min (E) and 50 min (F), respectively.

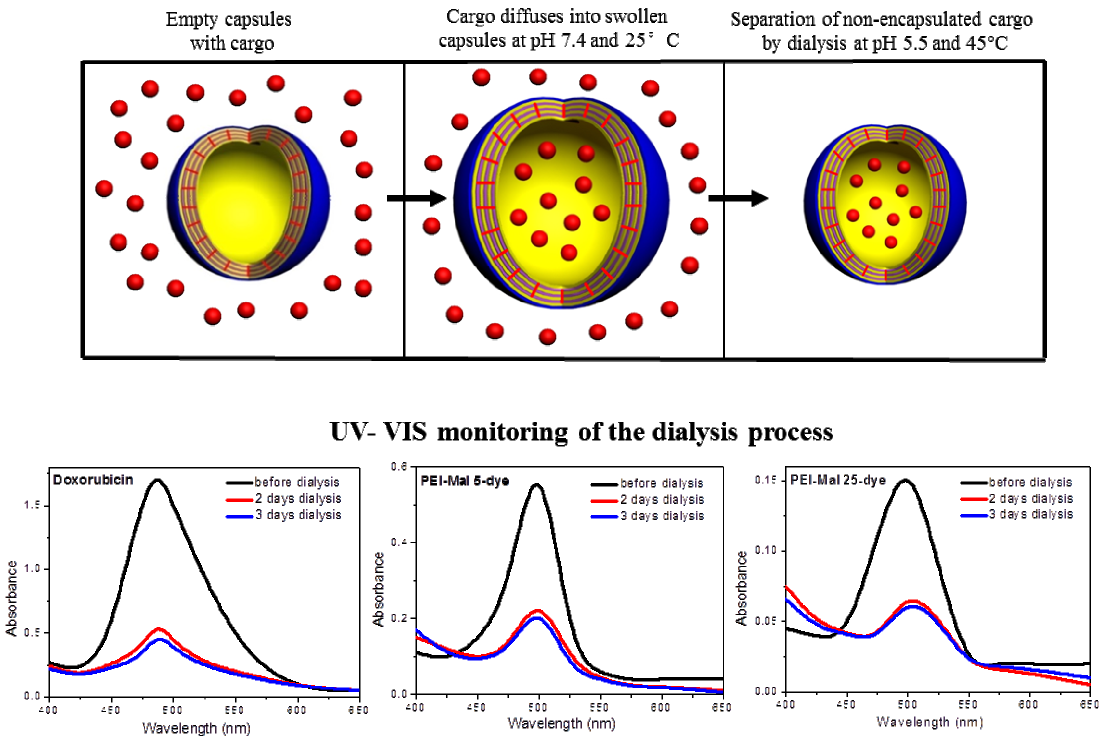

Figure S8. Schematic overview and UV-Vis monitoring how cargo is loaded into photo-crosslinked $[PNMB11/PAH]_3/PAH/PSS$ hollow capsules. Capsule membrane allows for transmembrane diffusion at pH 7.4 and 25 ℃. When cleaned afterwards at pH 5.5 and 45 ℃ using dialysis (dialysis tube MWCO 5000 for Dox and MWCO 150 kDa for PEI-Mal 5 and PEI-Mal 25, respectively), the Dox signal (bottom, left), rhodamine B labeled PEI-Mal 5 (bottom, middle) and PEI-Mal 25 (bottom, right) were still detectable and remained at a stable level, indicating a successful enclosure.

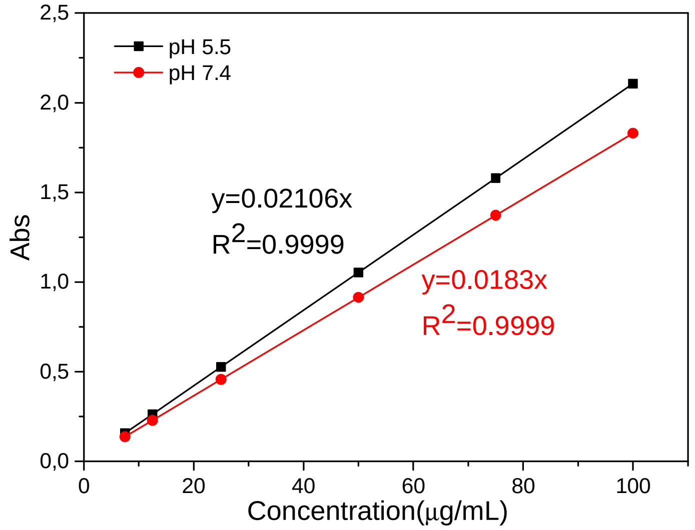

Figure S9. Calibration curves of Dox in phosphate buffer at pH 5.5 and in PBS at pH 7.4. The measurement was carried out at 488 nm using UV-vis spectrophotometer.

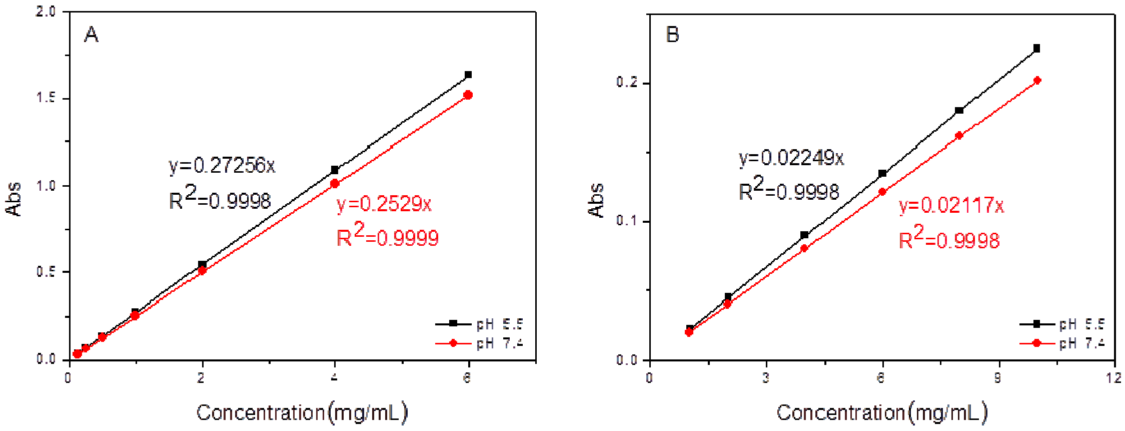

Figure S10. Calibration curves of the rhodamine B labeled PEI-Mal 5 (A) and PEI-Mal 25 (B) in phosphate buffer pH 5.5 and PBS pH 7.4. The measurement was carried out at 500 nm using UV-vis spectrophotometer.

3. REFERENCE

[1] D. Appelhans, H. Komber, M.A. Quadir, S. Richter, S. Schwarz, J. van der Vlist, A. Aigner, M. Müller, K. Loos, J. Seidel, K.-F. Arndt, R. Haag, B. Voit, *Biomacromolecules*, **2009**, *10*, 1114.

[2] S. Kunugi, Y. Yamazaki, K. Takano, N. Tanaka, M. Akashi, *Langmuir*, **1999**, *15*, 4056.

[3] H. Feil, Y.H. Bae, J. Feijen, S.W. Kim, *Macromolecules*, **1993**, *26*, 2496.
